# Hold the Gaba: A Case of Gabapentin-induced Hepatotoxicity

**DOI:** 10.7759/cureus.2269

**Published:** 2018-03-04

**Authors:** Christopher D Jackson, Michael J Clanahan, Kiran Joglekar, Sorawit T Decha-Umphai

**Affiliations:** 1 Internal Medicine, University of Tennessee Health Science Center Memphis; 2 Internal Medicine, Mercy Clinic Internal Medicine, St Louis, USA

**Keywords:** gabapentin, hepatotoxicity, drug-induced liver injury

## Abstract

A drug-induced liver injury is one of the most common causes of acute liver failure. While acetaminophen is the most common etiology, other offending medications include amoxicillin-clavulanic acid, amiodarone, isoniazid, and fluoroquinolones to name a few. Gabapentin, a gamma-aminobutyric acid (GABA) analogue, has infrequently been reported to cause liver injury; however, the causality in the previous reports is contested. Herein, we report a gabapentin-induced hepatocellular injury in a patient without another identifiable cause for acute liver injury. Discontinuing gabapentin resulted in rapid reversal improvement in hepatocellular injury.

## Introduction

Drug-induced liver injury (DILI) accounts for 10% of acute hepatitis cases annually [1]. The pattern of injury includes hepatocellular, cholestasis, or a mixed picture. DILI causes 20%-40% of fulminant hepatitis in the United States. While the most common cause is acetaminophen overdose, amoxicillin-clavulanic acid, isoniazid, trimethoprim-sulfamethoxazole, and fluoroquinolones are important considerations. Discontinuing the offending agent can often lead to reversal and improvement in hepatic injury.

## Case presentation

A 67-year-old African-American male with a history of type 2 diabetes mellitus complicated by neuropathy, hypertension, gastroesophageal reflux disease, and obstructive sleep apnea presented to the emergency room (ER) with chest pain. His chest pain was substernal in location, sharp in quality, and non-radiating. He had no previous history of myocardial infarction or congestive heart failure. His chest pain came on with exertion and was not completely relieved by rest or nitroglycerin. He denied fever, chills, sweats, shortness of breath, foreign travel, abdominal pain, nausea, or vomiting. His home medications included aspirin, vitamin D, docusate (as needed), gabapentin, lisinopril, loratadine, metformin, omeprazole, oxybutynin, sildenafil, and terazosin. He reported drinking one to two eight-ounce glasses of whiskey per night and occasional marijuana use. The physical examination was notable for normal S1 and S2 heart sounds, vesicular breath sounds, and a protuberant abdomen without evidence of ascites, hepatosplenomegaly, or other stigmata of chronic liver disease. He was given nitroglycerin and intravenous hydromorphone for pain. The electrocardiogram (ECG) obtained showed no ST-T changes concerning for acute ischemia. Laboratory studies were significant for two negative troponins, elevated aspartate amino aspartate (AST) and alanine aminotransferase (ALT), normal bilirubin, internalized normalized ratio (INR), and alkaline phosphatase (ALP) levels. Alcohol level was undetectable on admission. A review of the patient’s record shows he had normal transaminase, total bilirubin, and alkaline phosphatase levels one week prior (Table 1). On further questioning, the patient mentioned that he was started on gabapentin 300 milligrams twice a day exactly one week prior (05/19/2017) for diabetic neuropathy. He denied taking any new over-the-counter medications or herbs. Liver serologies on 05/19/2017 were obtained before gabapentin initiation.

**Table 1:** Temporal Changes in the Liver Serologies of Our Patient Aspartate aminotransferase (AST); Alanine aminotransferase (ALT); Alkaline phosphatase (ALP); Units per liter (U/L); Milligrams per deciliter (mg/dL)

Test (Units)	05/19/2017	05/25/2017	05/26/2017	05/27/2017	07/07/2017
AST (U/L)	28	576	639	242	28
ALT (U/L)	30	415	815	629	38
ALP (U/L)	79	111	137	149	89
Total bilirubin (mg/dL)	0.5	1.1	1.3	0.7	0.4

The patient was admitted to the internal medicine service for his chest pain and acute liver injury. The patient’s chest pain resolved in the ER after one-time administration of intravenous hydromorphone. Given the temporal association of the hepatocellular injury with the recent administration of gabapentin, the patient’s gabapentin was discontinued while he was an inpatient. The patient had a human immunodeficiency virus (HIV) and had an acute hepatitis diagnostic profile for A/B/C ordered, both of which were negative. Computed tomography (CT) of the abdomen and pelvis showed no evidence of cirrhosis or biliary obstruction. Mild hepatic steatosis unchanged from the previous examination was appreciated. The right-upper quadrant ultrasound showed no evidence of cholecystitis, and magnetic resonance imaging (MRI) showed no evidence of a gallbladder mass. The results of additional serologic testing for acute liver injury are presented in Table 2 to include thyroid stimulating hormone (TSH), creatine phosphokinase (CPK), total iron binding capacity (TIBC), and Epstein-Barr virus (EBV). The urine drug screen was positive for opiates (given in the ER) and marijuana. The transthoracic echocardiogram obtained showed no evidence of pericarditis, right ventricular systolic failure, or right atrial dilation.

**Table 2:** Laboratory Investigations for Acute Liver Injury Thyroid stimulating hormone (TSH); Micro international units per milliliter (uIU/mL); Creatine phosphokinase (CPK); Units per liter (U/L); Micrograms per milliliter (ug/mL); Total iron binding capacity (TIBC); Nanograms per milliliter (ng/mL); Cytomegalovirus (CMV); Enzyme immunoassay (EIA); Immunoglobulin M (IgM); Arbitrary units per milliliter (AU/mL); Epstein-Barr virus (EBV); Viral capsid antigen (VCA); Units per milliliter (U/mL); Human immunodeficiency virus (HIV)

Laboratory Study (Units)	Result	Reference Ranges
TSH (uIU/mL)	1.22	0.4 – 4.7
CPK (U/L)	90	20 – 320
Anti-mitochondrial antibodies (units)	8.1	0 - 20
Anti-smooth muscle antibodies (units)	20	0 - 19
Acetaminophen level (ug/mL)	< 10	10 - 20
Iron (ug/mL)	61	40 - 230
TIBC (ug/mL)	334	235 - 434
Ferritin (ng/mL)	23.4	10 - 320
Lipase (U/L)	131	20 - 200
CMV EIA IgM (AU/mL)	< 30	0 – 29.9
EBV antibody VCA IgM (U/mL)	< 36	0 – 35.9
Serum protein electrophoresis	No M spike observed	No M spike
HIV antibody screen	Negative	Negative or non-reactive
Hepatitis diagnostic profile (A, B, C)	Negative	Negative or non-reactive

Within 48 hours, the patient’s transaminase levels began to trend down. Serologic markers of hepatic synthetic function remained within normal limits. The patient was advised that his acute liver injury could be secondary to gabapentin, and he was instructed to discontinue taking gabapentin. At the outpatient follow-up with gastroenterology eight weeks later, the patient had a repeat testing of his transaminase levels, whose results were normal (Table 1). He continues to remain off gabapentin without any sequelae of liver disease.

## Discussion

Gabapentin, a gamma-aminobutyric acid (GABA) analogue with an obscure mechanism of action, is Food and Drug Administration (FDA) approved for the management of epilepsy and post-herpetic neuralgia [2]. Additionally, it is used, off-label, for the treatment of neuropathic pain due to diabetes mellitus. Gabapentin is not extensively protein-bound with its bioavailability most pronounced at lower dose levels [2]. Gabapentin has no appreciable liver metabolism, yet, suspected cases of gabapentin-induced hepatotoxicity have been reported.

Per literature review, two cases of possible gabapentin-induced liver injury have been reported. One case involved a 60-year-old male taking 2400 milligrams of gabapentin for chronic pain, who developed DILI with a mixed hepatocellular and cholestatic pattern [3]. The removal of gabapentin and the administration of steroids resulted in the normalization of liver test abnormalities. Another case reported a 26-year-old patient taking 1500 mg of gabapentin with associated hepatocellular injury [4]. In each case, however, the onset of liver test abnormalities occurred within one week of starting gabapentin. In the former case, there was a suspicion that the liver injury was more likely induced by ciprofloxacin, given the toxicoderma and cholestatic injury previously attributed to its use [5]. In the latter case, the attribution of the liver injury to gabapentin occurred without the documentation of workup for other causes of hepatic injury to include human immunodeficiency virus (HIV) infection or acute hepatitis. Additionally, the documentation of improvement in hepatic enzyme levels occurred 10 weeks after discontinuing gabapentin. Without further information, it is difficult to ascertain whether gabapentin was the causative agent of the patient’s liver injury and subsequent recovery.

In our case, gabapentin was felt to be the most likely etiology of acute liver injury for multiple reasons. First, there was a clear temporal association with the starting of gabapentin and the resultant transaminase elevation. Additionally, vigorous evaluation for other etiologies of liver failure did not suggest another cause to explain the acute transaminase elevation. Immediate and sustained decreases in transaminase levels were noted when gabapentin was discontinued. Moreover, the likelihood of DILI would be the highest with low-dose gabapentin administration given its higher bioavailability at lower doses. Although a liver biopsy was not pursued in this patient, we felt his overall clinical improvement obviated the need for this diagnostic modality.

## Conclusions

Gabapentin is an uncommon cause of DILI reported to cause a hepatocellular, cholestatic, or mixed picture of liver injury. Given the limitations of prior cases, we feel our report most closely ties gabapentin use to the resultant transaminase elevation. As in other cases of DILI, gabapentin-induced hepatotoxicity occurs within the first seven days post exposure to the drug. The discontinuation of gabapentin leads to an improvement in liver test abnormalities.
